# Assessing seal carcasses potentially subjected to grey seal predation

**DOI:** 10.1038/s41598-020-80737-9

**Published:** 2021-01-12

**Authors:** Abbo van Neer, Stephanie Gross, Tina Kesselring, Miguel L. Grilo, Eva Ludes-Wehrmeister, Giulia Roncon, Ursula Siebert

**Affiliations:** 1 Institute for Terrestrial and Aquatic Wildlife Research (ITAW), University of Veterinary Medicine Hannover, Foundation, Werftstraße 6, 25761 Büsum, Germany; 2 Present Address: CIISA-Centre for Interdisciplinary Research in Animal Health, University of Lisbon, Lisbon, Portugal; 3 Present Address: Institute for Marine and Antarctic Studies, University of Tasmania, Hobart, Australia

**Keywords:** Zoology, Animal behaviour, Behavioural ecology

## Abstract

In order to conduct an objective evaluation of potential ecological effects of grey seal predation on marine mammals, it is essential to establish a broad knowledge base helping in the thorough identification of such cases during post-mortem examination. The aim of this work is to report and discuss outcomes resulting from a retrospective evaluation of harbour (*Phoca vitulina*) and grey seal (*Halichoerus grypus*) stranding and necropsy data (n = 3274). In addition, the results are compared to a recent case of definite grey seal predation from Germany as well as reports from other countries. Carcasses potentially subjected to grey seal predation show severe lacerations with a circular pattern leaving a smooth, linear and cut-like wound margin. Large parts of skin and underlying tissue are detached from the body and loss of blubber is common. Occurrence frequencies of encountered lesions are presented and a list of parameters to be used for the assessment of similar cases as well as a complementary decision tree are suggested. With the proposed parameters, categories and tools, a baseline can be built in order to facilitate the standardised recognition of predation cases during post-mortem examinations of seals between groups working with populations across several geographic ranges.

## Introduction

Grey seals (*Halichoerus grypus*) are the most abundant seal species in the North Sea and in the majority of colonies their numbers are still increasing^1^. Survey data have shown that especially in areas like the Wadden Sea, where grey seals have been considered extinct mainly due to human exploitation until the late 1980s, their population is increasing^2^. In the German Wadden Sea the average increase of adult grey seals counted during moult surveys is 12% between the years 2006 and 2018, however harbour seals (*Phoca vitulina*) are still in the majority in this location^3–6^.

Besides the harbour seal and the harbour porpoise (*Phocoena phocoena*), grey seals are considered to be the top predator in this ecosystem. According to the literature, it is generally accepted that all three species of marine mammals mainly prey on fish^7–10^. When in 2012 and 2013 the first reports of suspected grey seal predation on harbour porpoises were published, it slowly became clear that the prey spectrum utilised by grey seals is more diverse than previously anticipated^11,12^. Initially, this concept was generally disregarded^13^, however, during the course of the following years more reports were published showing that grey seals not only prey on porpoises, but also on harbour seals and even regularly on individuals of their own species^14–16^. Recently, Brownlow et al.^17^ described cases from Scotland of juvenile grey seals that were observed to have been preyed on by a grey seal bull. Their gross pathological examination revealed traumatic lesions with detachment of skin and blubber, fractures and canine puncture wounds on the skull. Following a retrospective analysis of the local stranding database, grey seal bulls were assumed to be responsible for a considerable rate of seal mortalities in the area^17^. As a consequence of the fact that predation rates are largely unknown, the ecological significance of grey seal predation on marine mammals is still not yet understood and thus recognised entirely.

The main challenge remaining in this context is the diagnostic characterisation of wound patterns induced by grey seals and their differentiation from other wound patterns induced by traumata related to cutting (e.g. propeller strikes) or predation as well as post-mortem scavenging by other species, particularly foxes and birds. Due to the similarity of the lesions, there is the need for a standardised diagnostic protocol which utilises the existing knowledge to aid decision making.

Therefore, the aim of this study is to summarise the findings collected during necropsies of harbour and grey seals found dead between 1995 and 2018 on the coasts of Schleswig–Holstein and to suggest guidelines for future assessments of respective lesions. Results are discussed in comparison to a definite case of predation observed on Helgoland (Germany), as well as to cases reported in the literature and cases with other origins of trauma.

## Results

Carcasses of harbour and grey seals (n = 3274) have been recorded in the necropsy and stranding data base of Schleswig–Holstein, at the Institute for Terrestrial and Aquatic Wildlife Research (ITAW), University of Veterinary Medicine Hannover, Germany until the end of 2018 and are available for a retrospective evaluation. Of these 3,274 cases, 497 with records of external wounds were extracted and assessed further. Of the 497 cases, 207 were categorised as “unknown” and excluded from further consideration due to the lack of sufficiently detailed information or due to advanced decomposition. Of the remaining 290 cases, 97 were categorised as “observation only” and excluded from any pathological based assessment. This leaves in total 193 cases for the pathological assessment including the one definite case from 2018.

### Gross pathological examination

During necropsies of suspected cases of grey seal predation examined during the period 1995 to the end of 2018, different wound patterns have been recorded.

Based on our results, the most characteristic wound induced by grey seals is a large laceration with a smooth, linear, cut-like wound margin. The laceration often follows a helical course, starting in the area around the head and extending towards the caudal end of the body. Large parts of the blubber are usually detached from the underlying layer and manipulation of this tissue by teeth and claws is evident. The detached tissue can be turned inside out leaving the blubber exposed.

Using these and further findings, a catalogue of common parameters has been assembled to be used during macroscopic assessment of seal carcasses and is updated based on the existing knowledge and experience gathered (see Table S1 in the supplementary information).

**Figure 1:**
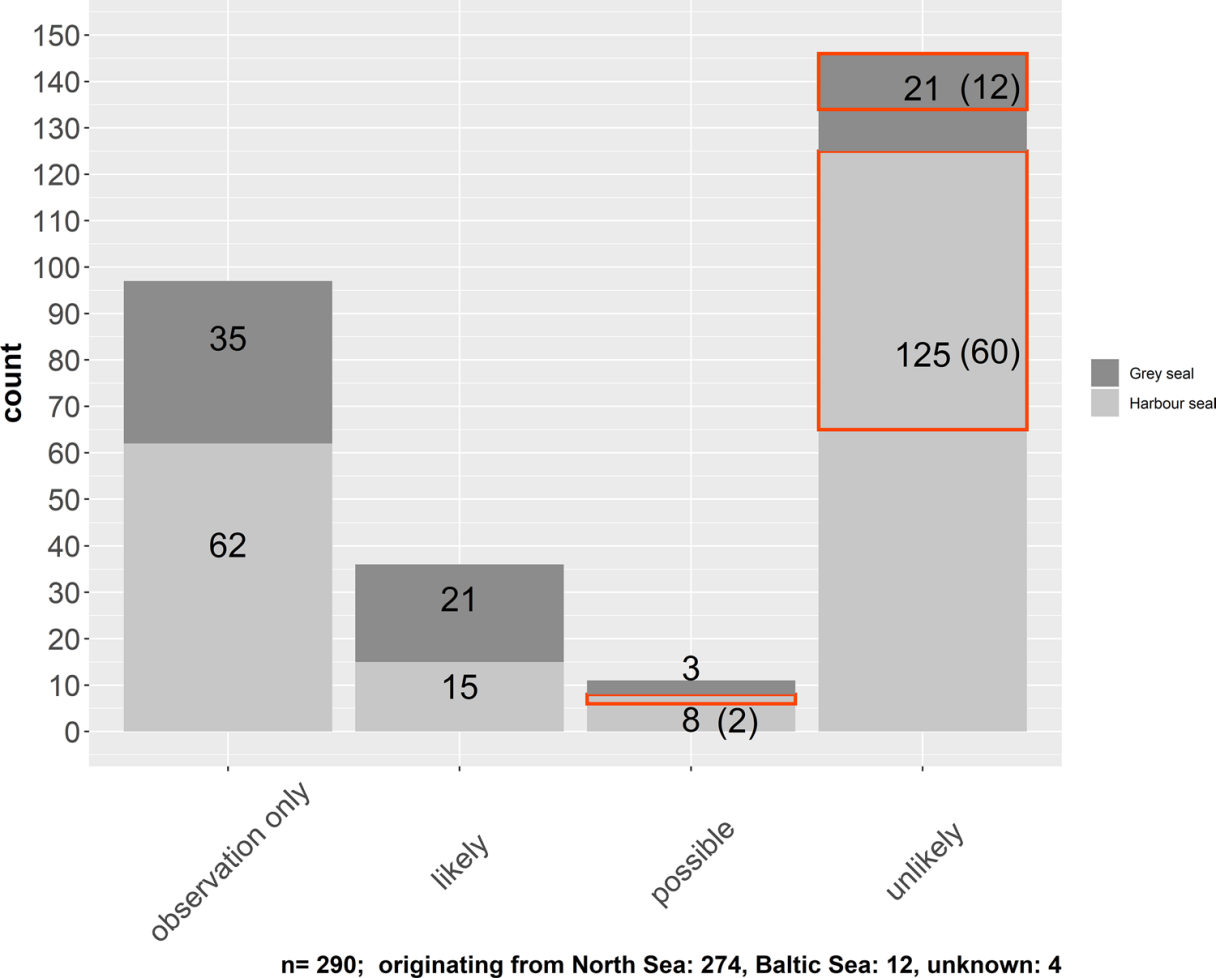
Number of suspected grey seal predation and fox related cases for the years 1995–2018; categorised by likelihood of grey seal predation (“likely”, “possible”, “unlikely”), cases that have only been observed but without recovery of the carcass (“observation only”) and suspected fox interactions. Cases related to fox interaction were included in the category “unlikely” and possible in terms of grey seal predation and are highlighted by a red square. The number is shown in brackets. The definite case from 2018 is included as likely in this figure. Number of grey seal victims are shown in dark grey, number of harbour seal victims in light grey bars.

**Figure 2:**
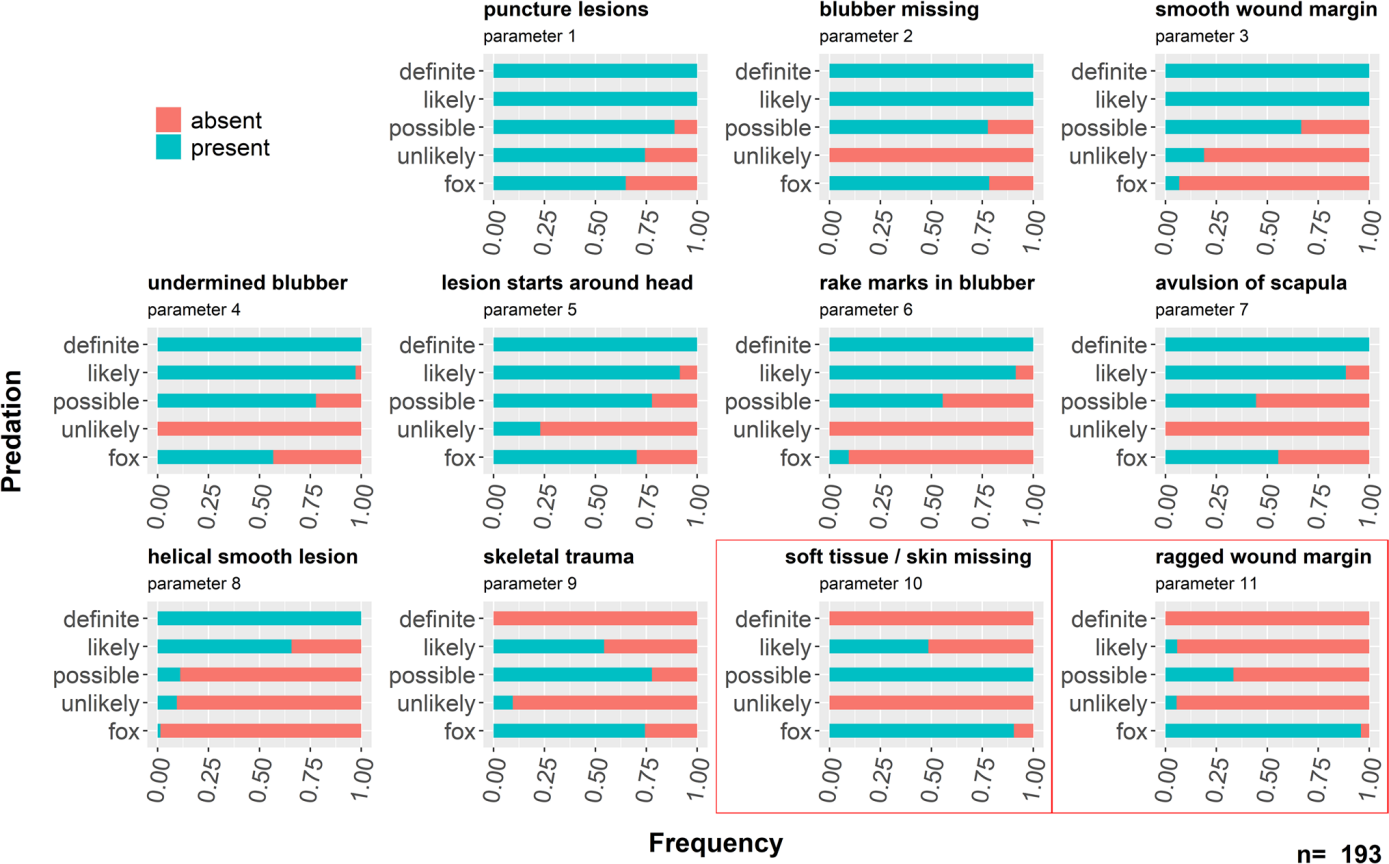
Percentage of occurrence of the 11 parameters in the different categories for suspected grey seal predation cases (“definite”, “likely”, “possible”, “unlikely”) and for suspected fox interactions (“fox”). Cases related to fox interaction are only shown in the category “fox”, despite also being “unlikely” with regards to grey seal predation. Parameters framed with a red rectangle are indicative for an interaction with a fox.

Using the absence and presence of respective parameters, 193 cases of suspected grey seal predation were judged and assigned to either of the categories “unlikely”, “possible”, “likely” or “definitely” (for the definition of categories, refer to Table 1) respectively (Figs. 1, 2). Analysis of possible fox interaction was also included.

**Table 1:** Categories and their respective description used for rating the likelihood of grey seal predation and fox interaction.

Category	Definition
Definite	The attack was observed and the carcass was retrieved straight after
Likely	It is highly likely that the documented trauma is the result of grey seal predation; the majority of parameters 1–9 have been found
Possible	It is possible that the documented trauma is the result of grey seal predation; some of the parameters 1–9 have been found; potentially some indication of a different origin of trauma
Unlikely	It is unlikely that the documented trauma is the result of grey seal predation; the majority of parameters have not been found; clear indication of a different origin of trauma
Fox	It is unlikely that the documented trauma is the result of grey seal predation, but indicators of an interaction with a red fox have been found (parameters 10, 11)

### Decision tree

**Figure 3:**
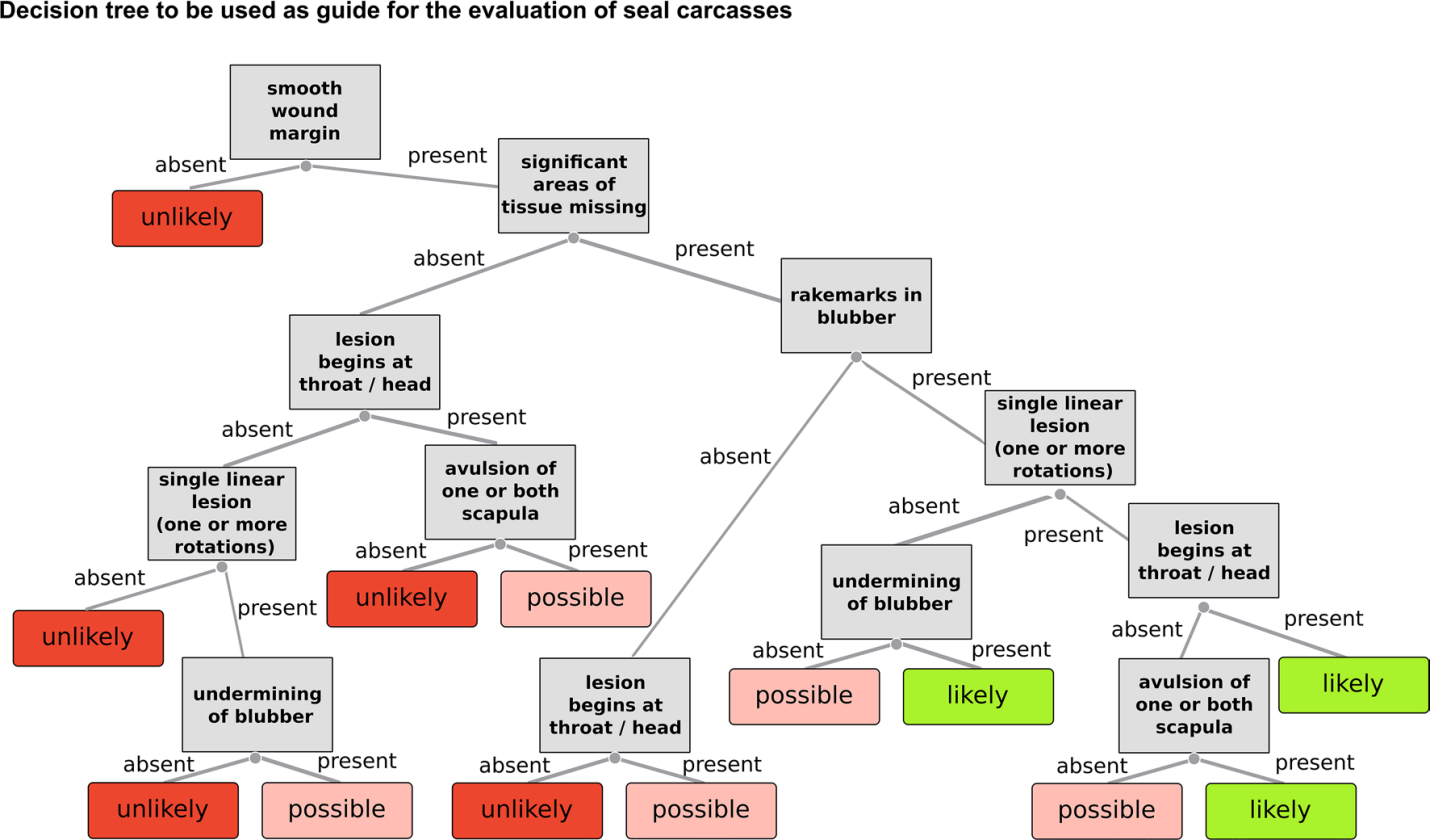
Decision tree developed using the occurrence and combination of detected lesions in seal carcasses. Resulting categories indicate the likelihood that the detected lesions are the result of grey seal predation.

Using the presence or absence as well as the combination of suggested parameters in conjunction with the rating of the likelihood of grey seal predation, a decision tree was developed to be used as a guide during the decision making process (Fig. 3).

In order to verify the accuracy of the decision tree, all cases were re-analysed solely using the tree. These results in turn were compared to the rating given by the expert. Of the 193 cases suspected to be the result of grey seal predation, the tree rated the likelihood in 95% of the cases in the same way as the expert. Cases rated differently often showed an inconsistent pattern of present lesions and the decision by the tree was in all but one case a more precautious judgement (e.g. instead of rating the case with “possible”, “unlikely” was suggested).

### Anecdotal data

Additionally to the cases for which a carcass was retrieved, we filed 97 cases of anecdotal reports of suspected grey seal predation of seals which have been recorded by professionals and semi-professionals in the field (Fig. 1—category observation only).

## Discussion

The phenomenon of grey seal predation of marine mammals has only recently been described in more detail in the scientific literature^14,15,17,18^. Yet, the increasing numbers of reports need to be seen as an indicator for the potential ecological relevance of this behaviour. Therefore, in order to gain a better understanding of the scope and thus relevance of this behaviour, it is essential to quantify the number of cases applying standardised assessment criteria.

The results presented here summarise the findings of necropsies conducted on 193 harbour and grey seals from the coasts of Schleswig–Holstein suspected to have been preyed on by a grey seal. The respective 11 parameters have been developed during necropsies over the last years and represent the most common lesions recorded.

Most evident in seal carcasses subjected to grey seal predation is a large tissue defect of the epidermis and the underlying blubber with a long, linear wound margin resulting from the tearing of the skin (parameter 3, 8). Further epidermis and blubber are most often partly detached (parameter 4). In this way, the predator gains access to the blubber tissue rich in caloric content which is then subsequently removed from the skin using the teeth. The scraping off the blubber usually leaves a distinct uneven pattern as well as typical rake marks in the blubber tissue (parameter 2, 6)^18^. Lacerations induced by tearing of the skin show a cut-like, smooth and linear wound margin (parameter 3) which often starts in the cervical area (parameter 5) and follows in a helical manner around the body (parameter 8). Furthermore, puncture wounds potentially induced by the intrusion of the teeth, particularly canines can be found in the skin / blubber (parameter 1) and bones (parameter 9). The avulsion of one or both scapulae (parameter 7) has been commonly documented and is potentially the result of the force applied when detaching the epidermis and blubber from the muscular tissue of the prey.

Looking at the frequency of the parameters described (Fig. 2), several qualify as good indicators in order to assess the likelihood of grey seal predation as an origin of trauma. Parameters 1–7 show very high (> 85%) and parameter 8 high (> 65%) occurrence frequencies in “likely” cases and could also be confirmed as “present” in the definite case^18^. While they show a low prevalence in unlikely cases. It is noteworthy, that some variation is evident as some parameters, despite being found regularly in grey seal predation cases, can also be caused by other means such as scavenging by other predators (e.g. puncture lesions (parameter 1) or missing blubber (parameter 2)). Parameters 8–9 show a lower frequency for the “likely” cases with the parameter “skeletal trauma” occurring least frequently (54%). The latter can be explained by using the observational reports. Grey seals seem to mainly target the blubber as a source of high energy content by detaching the skin from the body^18^. During this process, the skeletal parts of the body are usually not manipulated with exception of the scapulae. If present, skeletal trauma is also often found in the area of the skull possibly resulting from catching and retaining the prey, which has been described before^17,18^. Overall, skeletal trauma as well as puncture lesions cannot be considered as a very good indicators for grey seal predation as they can have many different natural and unnatural aetiologies. Nevertheless, they should be used as additional indicators, especially when there is the possibility to measure the inter-canine distance which can be indicative for a specific predator. In contrast, the avulsion of one or both scapulae (parameter 7) and a smooth, cut-like lesion following a helical course around the body (parameter 3 and 8) can be seen as typical, even if they are not detected in every single case.

Loss of muscle tissue can most likely in large parts be attributed to scavenging e.g. by birds. This is supported by the definite case^18^, as well as an earlier observation made by the authors in 2016 on Helgoland, where a potentially predated harbour seal showing the typical lesions was washed ashore and the muscle tissue in the area of the wound, which was largely intact at the time of the stranding, was removed considerably by birds within 90 min.

As has been described in the literature before, e.g.^18^, assessing the condition of the hair bordering the wound margin on the cranial side of the laceration can give first important indications if the tissue defect was induced by cutting or shearing forces and thus, should be considered in any future assessments. As this factor has not been verified throughout all necropsies reported here, it was excluded from the list of parameters but should be added to the list and used in future assessments (for the updated version of parameters see Table S1 in the supplementary material).

When assessing seal carcasses in the context of grey seal predation, which were retrieved from areas with terrestrial predators and/or scavengers present, it should be noted that lesions induced by terrestrial mammals are often difficult to differentiate from the ones induced by grey seals. On the German island of Sylt, several carcasses have been retrieved also showing extensive defects of the skin and the underlying tissue. But in contrast to Helgoland, where no large terrestrial predators occur, these cases have been confirmed as either predation (confirmed by personal observation through the local seal ranger) or most likely post-mortem scavenging (documentation of pugmarks and other indicators surrounding the carcass) by the red fox (*Vulpes vulpes*). In order to differentiate between terrestrial and marine mammal feeding, again the wound margin should be assessed carefully. As can be seen in Fig. 2, in grey seal predation a linear and fully smooth wound margin is considered typical (parameter 3, 8). In cases of feeding by foxes, a ridged wound margin (parameter 11) with considerable areas of missing soft tissue (parameter 10) can be expected. This potentially originates from the separation of the tissue by using their carnassial teeth, opposed to tearing as seen in grey seals. Further, the location and course of the wound can be indicative. Wounds produced by grey seals most often start in the area around the head (parameter 5) and follow a rather helical course (parameter 8) often with the possibility of fully repositioning both sides of the wound margin. In contrast, in lesions induced by foxes, most often vast amounts of skin are missing in different areas of the body and wounds resemble areas where most soft tissue has been removed and only the bones are left (parameter 10). In addition to the general macroscopic assessment, wound margins on the cranial side of the lesion should also be assessed to check for an increased degree of damaged hair shafts which would also be expected in cases of scavenging by terrestrial mammals.

Lesions presented here are consistent with the results reported by Brownlow et al.^17^ and also with an earlier report on grey seal predation of harbour seals from Helgoland^15^. Therefore, the resulting parameters should be seen as an important set of indicators when it comes to evaluating the cause of trauma (*intra vitam*) or scavenging (*post-mortem*). Due to the possibility of other factors causing respective alterations, it needs to be emphasised that no single parameter should be used alone in order to rate the likelihood of grey seal predation. This holds especially true for areas with known occurrence of other marine and / or terrestrial predators known to utilise seals as prey resource. This becomes evident when comparing different descriptions of potentially shark inflicted lesions in seals. Lesions induced by great white sharks (*Carcharodon carcharias*) for example, have been described for numerous seal species often resembling crescent shaped lesions bearing a ragged wound margin and often multiple incisions by the teeth as well as in some cases shredded skin hanging from the remaining tissue^19–21^. Other sharks known to feed on seals are comprised of different members of the family Somniosidae (sleeper sharks)^22^. These sharks have been described to also inflict rather characteristic wounds in seals, with crescent to circular shaped lesions^23^ presumably originating from a bite and turn behaviour (only known recording of such behaviour can be seen in the BBC documentary Blue Planet 2). In contrast to great white sharks though, sleeper sharks leave a comparably shallow, smooth edged wound^23^. Wounds inflicted by grey seals differ in comparisons to wounds induced by great white or sleeper sharks, as they often show a largely intact coat, which is despite being regularly turned inside out (parameter 4), often only separated in a single helical laceration (parameter 8). Further, in grey seal induced lesion, often only the blubber is removed (parameter 2), whereas skin and muscle tissue is not significantly removed in most cases (cf. Fig. 2, parameter 10) which is in contrast to shark induced lesions^19–21^. The importance of such differentiation becomes evident when for example comparing the results of this study with cases from Sable Island, Canada. On Sable island, the death of over 4000 seals has been attributed to Greenland sharks (*Somniosus microcephalus*)^24^, despite the fact that this species has been shown to be a very slow swimming fish presumably only capable of catching sleeping or deceased seals^25^, as well as that the lesions of the shown cases bear a striking similarity to the ones described here and in prior publications^15–18^.

Further known causes of trauma in seals is the predation by orcas (*Orcinus orca*)^26^. Whereas for grey seal predation it is not known that the majority of the preys’ body is consumed, in killer whale predation this is most often the case^26,27^. For cases which have presumably escaped predation attempts by orcas, parallel running lacerations as well as puncture lesions with inter-teeth distances not attributable to grey seal predation have been described^23,28^.

In contrast to the rather well distinguishable sources reported above, anthropogenic induced lesions can proof more difficult to distinguish, as lesions induced for example by propeller strike can show similarities to grey seal induced lesions. The difficulty in the differentiation is mainly due to the also possible helical and smooth edged nature of propeller wounds^17,29^. In such cases, especially the presence of damaged hair shafts (see parameter 12 in the supplementary material) can aid during the decision making process, but also parameters with reference to feeding such as the removal and structure of the blubber (parameters 2, 6). In general it needs to be emphasised, that there can be many different and often regionally specific causes of trauma in pinnipeds which need to be considered during an assessment of a suspicious case.

Overall, only the combination of parameters in conjunction with any other information available (e.g. information on the stranding site (including known occurrence of predators/scavengers) and situation) allows for a thorough evaluation of the potential cause of death. Yet, a definite proof that grey seal predation is the origin of a trauma in a seal carcass can, to date, only be based on an observation.

In order to further facilitate a standardised assessment protocol of suspected cases, a decision tree was developed using the data presented here. The comparison between the results generated by the tree and the expert indicates, with an accuracy of 95%, that the tree can well be used as a further guide during the decision making process. It needs to be emphasised though, that due to the complexity of the subject and the limitation of the used data set, the suggested decision tree should only be used as a guide. In order to maximise the accuracy of the result, all tools available should be considered and the final decision needs to be exclusively based on the evaluation of all available information conducted by the responsible expert.

Up until today, several observational/anecdotal reports of grey seals preying on marine mammals have been filed in Germany (Fig. 1). Such reports originate from different groups ranging from the general public, through semi-professionals to professionals in the field of marine mammal science. Taking into account such information, as well as detailed accounts of behavioural observations as recently described in the literature^16–18^, the behaviour seems to follow fairly stereotyped steps. Considering the reported observations, most parameters suggested above can be linked to a specific behaviour.

**Figure 4:**
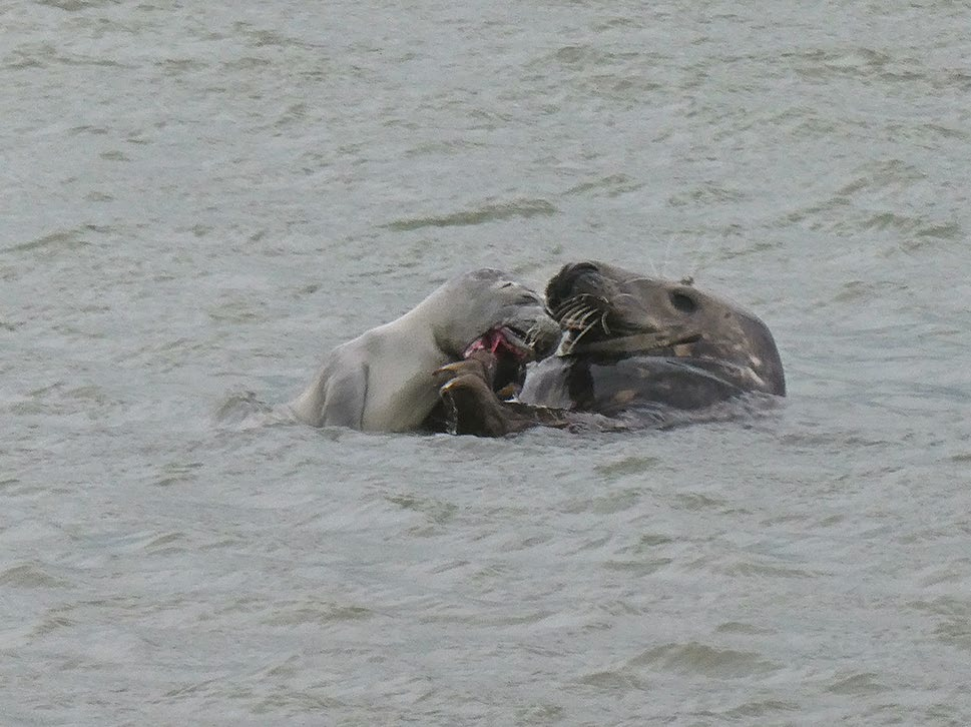
Subadult grey seal bull with a juvenile harbour seal as prey and shown while inducing the typical helical lacerations by tearing apart the tissue using its pectoral flipper and jaws. Helgoland, October 2017. Picture courtesy of Susanne Hauswaldt.

Following the death of the prey (usually either by exsanguination or by asphyxiation), the carcass is opened near the neck and throat area (parameter 5, see also Fig. 4) using the pectoral flippers and jaws. The tearing of skin leaves a smooth, linear, cut-like laceration (parameter 3, 8). It also results in the detachment of the blubber from the body (parameter 4) and can also cause the avulsion of one or both scapulae (parameter 7). Blubber is scraped of the detached areas of skin using the teeth (parameter 2, 6) and biting can induce puncture lesions throughout the carcass (parameter 1).

**Figure 5:**
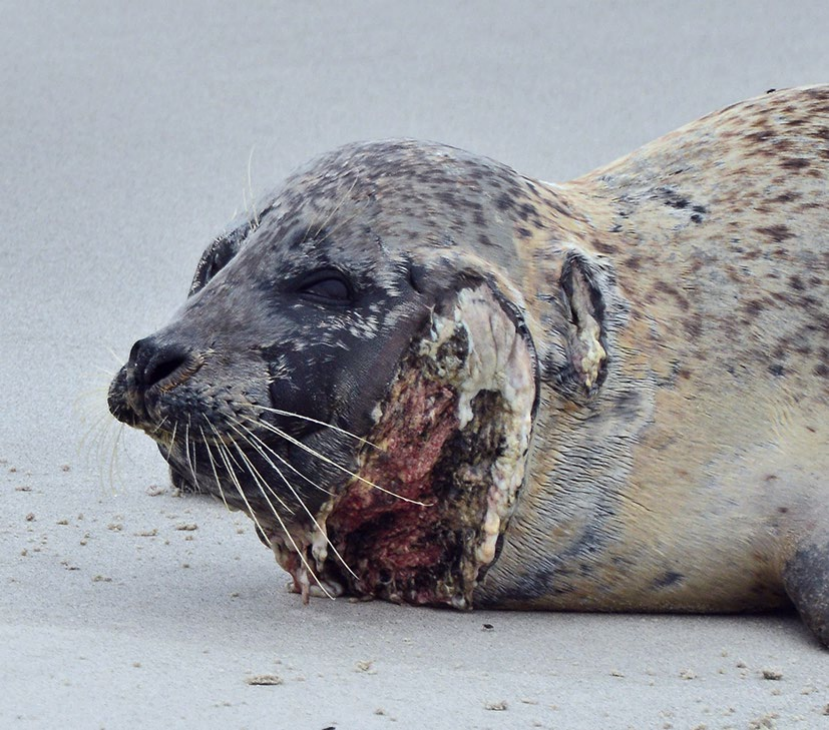
Harbour seal with severe injuries and infections in the area of the throat and head. Helgoland, Germany, September 2018.

For porpoises, it has been described that individual animals most likely have escaped attacks by grey seals and apparently survived the inflicted injuries at least for some time^30^. For seals, this is barely documented. One potential case of a harbour seal has been documented on Helgoland. As can be seen in Fig. 5, the seal showed severe injuries in the area of the throat which could potentially be the result of a prior grey seal attack. Nevertheless, despite the similarity of lesions, other sources of trauma cannot be excluded.

To date, only subadult or young adult male grey seals have been documented showing this behaviour in Germany. Until now, at least four different individuals have been identified to prey on seals, by means of pelage pattern or flipper tag ID. They have been observed to prey on harbour seals as well as grey seals, but most often with the prey not older than two years. The oldest animal observed to have been preyed on was a female grey seal judged by the outer appearance to be three to four years of age. One predating bull was observed showing this behaviour over consecutive years, as well as utilising grey and harbour seals as prey resource on separate occasions (see report in van Neer et al.^18^ and Fig. 4, both describe / show the same individual bull). This indicates that such specialisation is not necessarily focused on a single prey species but potentially directed towards marine mammals as prey resource in general. It is further unclear, if single individuals utilise both, seals and porpoises, as so far no single individual has been reported preying on seals and porpoises alike. In order to verify this, genetic analysis of e.g. scat samples as well as further observational studies should be conducted.

## Conclusion

In order to establish a reliable diagnostic procedure for the differentiation of potential causes of lesions on carcasses of marine mammals with regard to grey seal predation, it is crucial to examine any carcass carefully and in detail using standardised parameters like the guidelines presented in this study. Additionally, any source of information like reports from the site of the stranding or eyewitness reports should be used in the decision making process. Further, the use of the decision tree presented here is recommended as an additional tool. Nevertheless, the final decision should always be made in consideration of all available information. Parameters should also be continuously refined based on any new evidence that arises. Further research should be conducted in order to assess the suitability of other methods that could be used in the differentiation process, such as the histological evaluation of wound margins to differentiate between causes of trauma (e.g. cutting or tearing) or easy to use methods enabling the detection of grey seal DNA in harbour seal carcasses similar to the ones suggested for porpoise carcasses^31,32^.

Cases of observed grey seal predation should be further used as reliable source of information with regards to the behaviour as well as pathology. Scientists working on this topic should continue collaboration in order to constantly update and improve the list of pathological parameters and assessment tools. Also, if possible, research groups should work together in establishing an objective method and thus allow for a comprehensive evaluation of the potential ecological effects of grey seal predation on marine mammals in the future.

## Methods

Carcasses were collected through the local stranding network in Schleswig–Holstein, Germany^33,34^ and a necropsy was conducted^35^. In addition, one well documented case from 2018^18^ was taken for validation of recorded wound patterns. As part of the necropsies, age was estimated, sex was determined, weight, length and other measurements were taken and the health assessed and cause of death judged. Due to the differences in the decomposition state of the different parts of the body of predation cases, decomposition state of the animal was rated by assessing the remaining intact parts of the body. This gives a more realistic picture as the manipulated parts show an advanced state of decomposition due to the direct exposure to the environment. In most cases, detailed information on the stranding site, condition of the carcass when found, as well as any other useful information was recorded by local volunteers.

Of the 3274 stranding records available, cases of 404 harbour seals and 93 grey seals with any recorded external wound were rated as suspicious and selected for further investigations with regard to potential grey seal predation. Results were taken from the necropsy and stranding database of the Institute for Terrestrial and Aquatic Wildlife Research (ITAW), University of Veterinary Medicine Hannover (Büsum, Germany) and evaluated retrospectively.

Cases which were considered too decomposed for a thorough evaluation or data deficient were categorised as “unknown” and excluded from the study.

Since the recognition of the predation phenomenon and the start of a specific research project in 2015, a detailed protocol was developed for recording potential cases of grey seal predation as part of the necropsy (see Figure S1 for the updated version based on the results presented here).

Using necropsy results, existing pictures as well as any additional information available, cases were judged and rated in five prior defined categories (definite, likely, possible, unlikely and fox) by experienced scientists depending on the likelihood of grey seal predation as an origin of documented lesions (Table 1). Further, the frequency of recorded lesions was determined and based on these results, parameters for future pathological assessments were constituted.

Following the subjective judgment and respective rating into one of the prior defined categories, results were used to develop a decision tree based on the occurrence and combination of the suggested parameters (Fig. 3). In order to verify if the decision tree rates given cases correctly when compared to the expert opinion, all cases where re-analysed solely based on the decision tree and results were compared accordingly.

Besides any cases where the carcass was retrieved and was available for necropsy, anecdotal data consisting of well-documented observational reports from Helgoland mostly recorded by professionals and semi-professionals and for which no carcass was retrieved, have been judged by an experienced researcher and filed and summarised respectively. These cases have been labelled “observation only”.

### Ethics approval and consent to participate

Carcasses used in this study were forwarded to the Institute for Terrestrial and Aquatic Wildlife Research for necropsy as part of the national stranding network and monitoring scheme. As all research was conducted on deceased animals, no ethics approval was needed.

## Supplementary Information

Supplementary Information

## Data Availability

All data generated or analysed during this study are included in this published article [and its supplementary information files].
