# Supplementary material for: Assessing seal carcasses potentially subjected to grey seal predation
Source: Sci Rep. 2021 Jan 12;11:694. doi: 10.1038/s41598-020-80737-9 (PMC7804150; doi:10.1038/s41598-020-80737-9)
Supplement: Supplementary file 1 — Supplementary Information [file 41598_2020_80737_MOESM1_ESM.pdf]

Assessing seal carcasses potentially subjected to grey seal predation

Authors:

Abbo van Neer, Stephanie Gross, Tina Kesselring, Miguel L. Grilo, Eva Ludes-Wehrmeister, Giulia Roncon, Ursula Siebert

Supplementary Information

Table S1: Table showing 12 parameters, which have been added to the catalogue and are now routinely used for rating the likelihood of grey seal predation as origin of a trauma in seals. Pictures for parameters 1 - 8 courtesy of Dominik Nachtsheim.

**1**	**Puncture lesions:** Puncture lesions are present in the skin or blubber tissue.	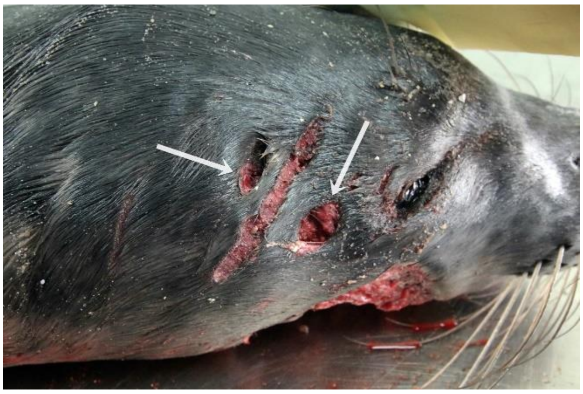
**2**	**Missing of blubber tissue:** Considerable parts of the blubber tissue show signs of manipulation. Often a diffuse uneven blubber surface with an irregular blubber depth (slightly roundish structures surrounded by areas of reduced blubber depth) is evident. Blubber depth along the fringes of the skin flaps is in parts reduced and less than towards the middle areas.	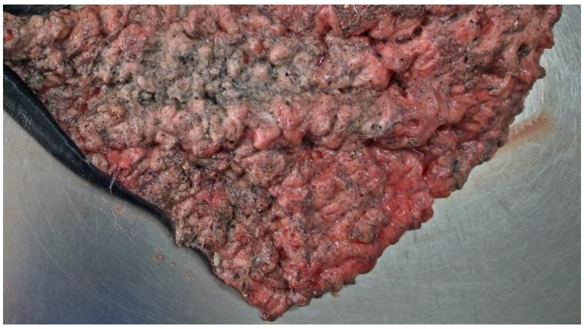

**3**	**Smooth wound margin:**<br>Due to the tearing of the skin, a smooth, linear, cut-like wound margin is present throughout large parts of the lesion.	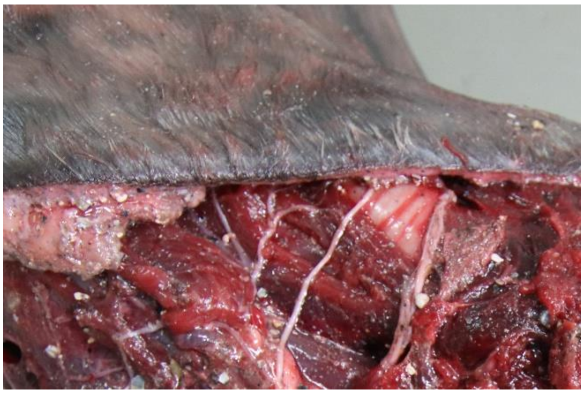
**4**	**Undermining / detachment of blubber:**<br>Considerable parts of the blubber are detached from the underlying muscular tissue in large parts of the body area. Only in the areas around the caudal end of the body, the pectoral flippers, as well as the rostral part of the head, the skin and underlying tissue is often still attached.	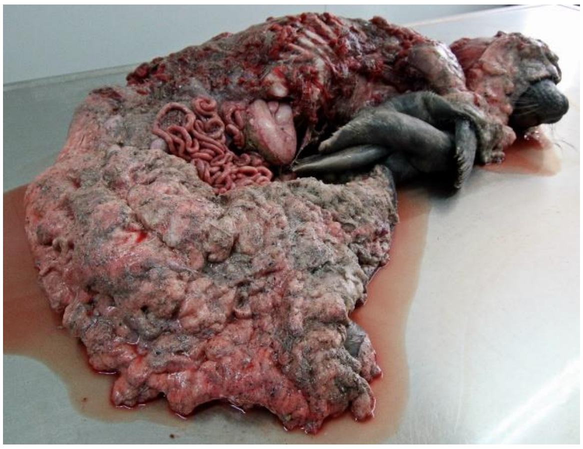
**5**	**Start of lesion in throat / head area:**<br>The origin of the lesion lays on the ventral side of the neck or around the lower jaw / throat area, pieces of skin including the blubber can be missing in this part.	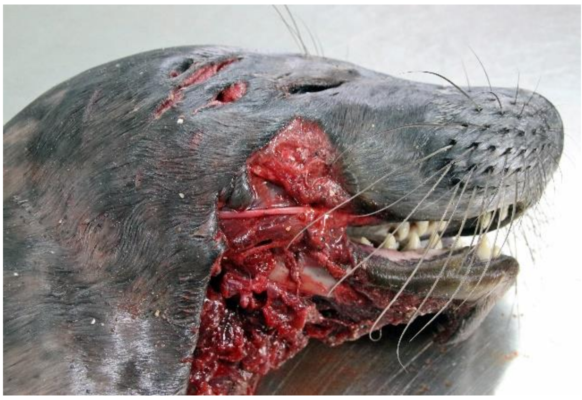

**6**	**Rake marks in blubber:** Rake marks potentially as the result of the incisions by the teeth and / or claws are present in parts of the blubber tissue.	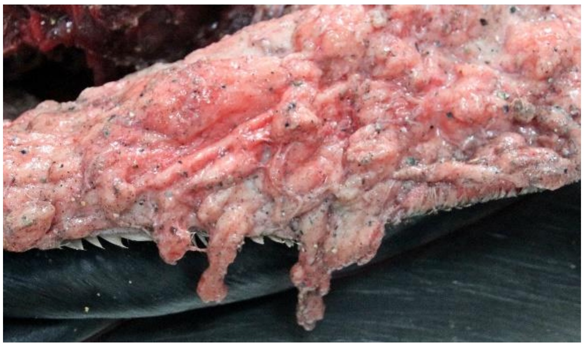
**7**	**Avulsion of one or both scapulae:** An avulsion of one or both scapulae due to the detachment of the skin and blubber (including the pectoral flippers) can be present.	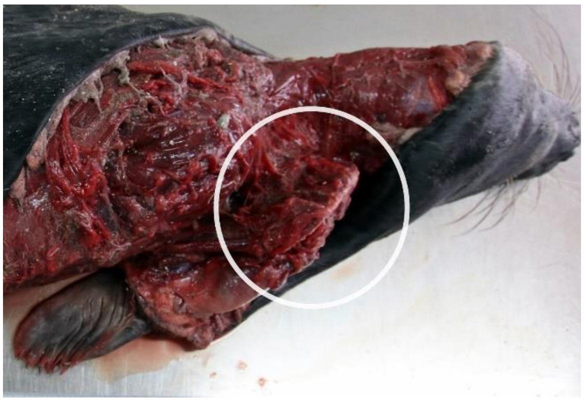
**8**	**Helical smooth edged lesion:** A smooth edged laceration which follows a helical course, starting in the area of the throat / head and circling backwards once or twice around the body is a common pattern observed.	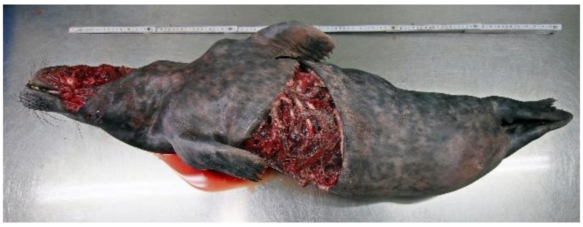
**9**	**Skeletal trauma:** Fractures of bones with different severity can be present. Fractures of parts of the skull have been observed. Also puncture like fractures in the lower mandible and / or scapula have been observed commonly.	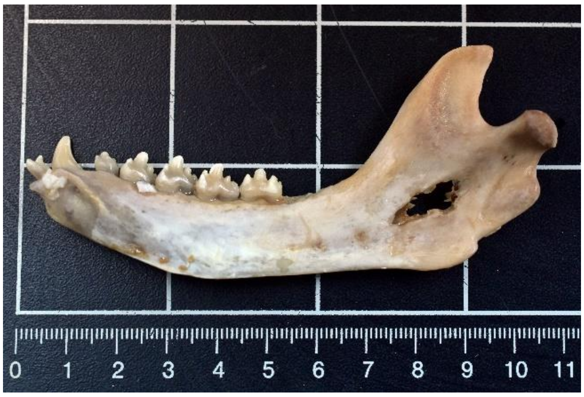

**10**	**Vast amounts of soft tissue and skin are removed:**<br>Lesions which can be situated anywhere on the body with vast amounts of skin, blubber and muscle tissue missing are especially in addition to a ragged wound margin a strong indicator for an interaction with a red fox.	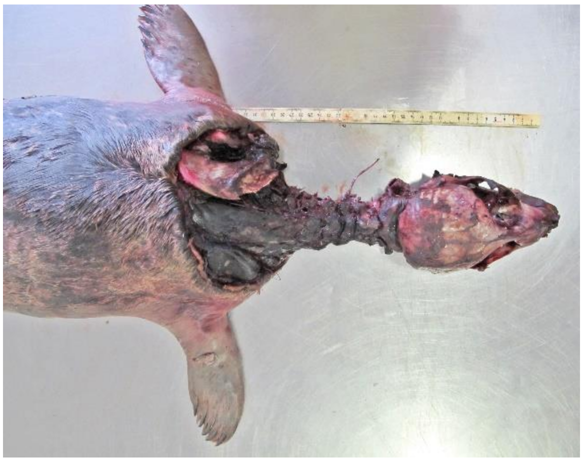
**11**	**Ragged wound margin (fully or in parts):**<br>Considerable parts of the wound margin have a ragged, uneven and not cut-like structure. This is a strong indication for an interaction with a red fox.	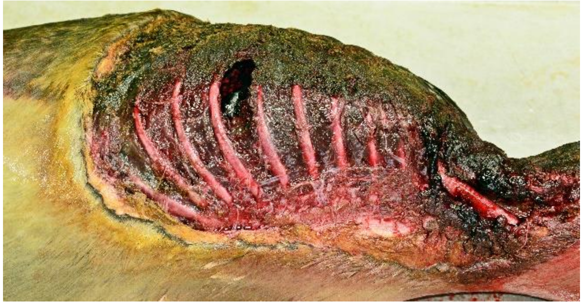
**12**	**Defect of hair shafts:**<br>In contrast to torn tissue where on the cranial side of the linear wound margin largely intact hair extends over the edge of the tissue, in cut tissue the extending hair on the cranial side often shows structural damage of the hair shafts.	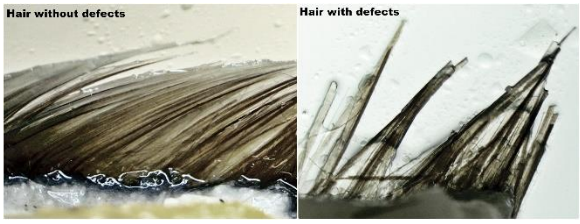

Figure S1: Newly developed protocol to be used for the documentation of suspected grey seal predation cases in its updated version based on the results presented here.

PINNIPEDIA-PROTOCOL

Team:…………………………………………

Prep-No................................ **Species**....................................... total length.................. cm

Date of retrieval........................... Prep-date............................ weight.......................... kg

Place of stranding.. Nr........................ sex m ◯ f ◯

Retrieved by...................................... Condition during delivery ☐ fresh ☐ frozen

Transponder / Tag number...

estimated age ◯ juvenile ◯ subadult ◯ adult

Decomposition status ☐ (1-5)

Stranding Report

Pictures from stranding site: O yes O no

Animal tracks on or near site of stranding?

O terrestrial predator/scavenger O seal O none O no information

Prep.-No:________________________

Documentation of lesions and sampling sites:

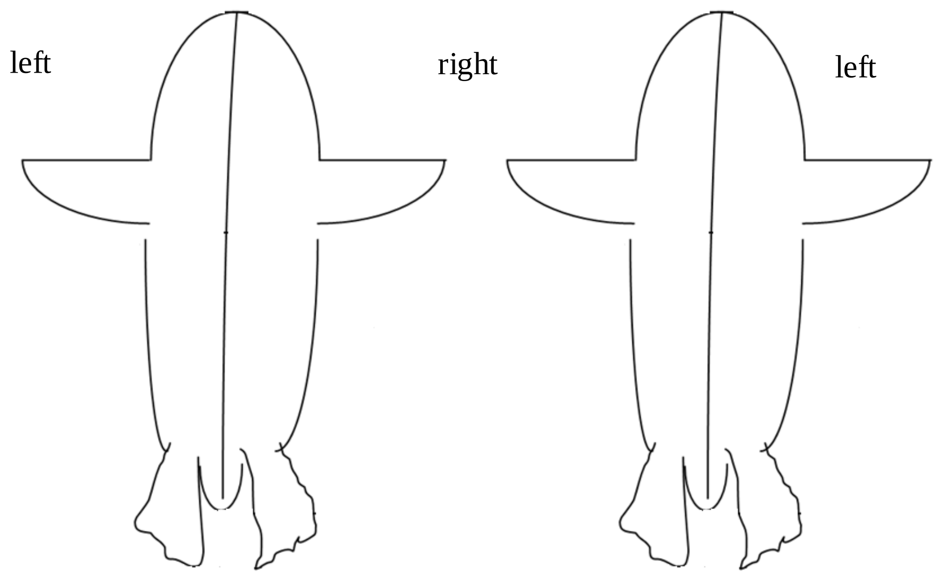

dorsal ventral

Right side of body:

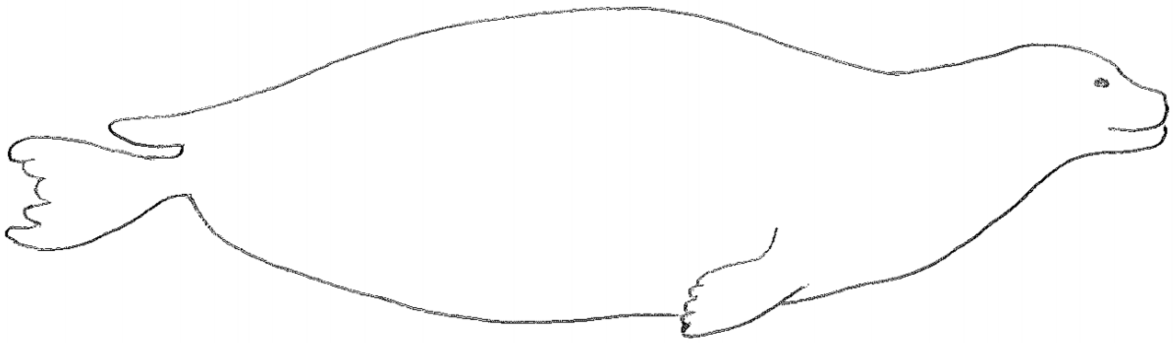

Left side of body:

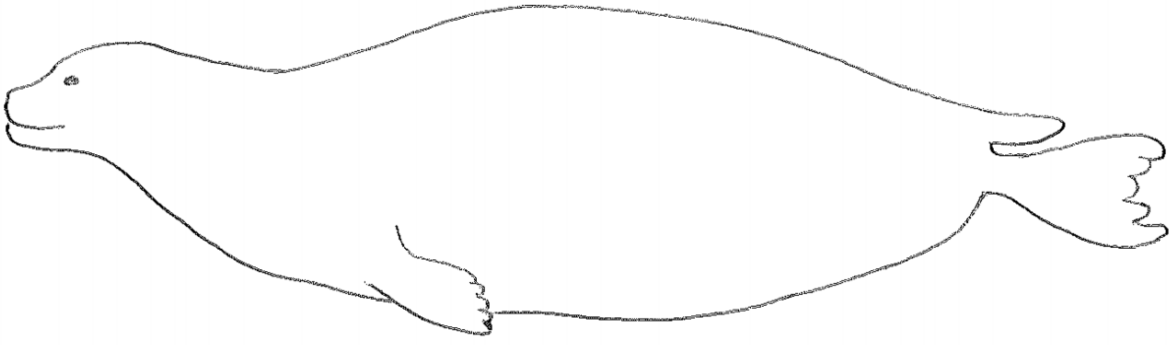

Prep.-No:______________________

Occurrence of Parameters:

No	Parameter	Absent	Present
1	Smooth wound margin: Due to the tearing of the skin, a smooth, cut like wound margin is present throughout large parts of the lesion.	☐	☐
2	Missing of Blubber tissue: Considerable parts of the blubber tissue show signs of manipulation. Often a diffuse uneven blubber surface with an irregular blubber depth (slightly roundish structures of increased blubber depth, surrounded by areas of decreased blubber depth) is evident. Blubber depth along the fringes of the skin flaps is in parts reduced and less than towards the middle areas.	☐	☐
3	Start of lesion in throat / head area: The origin of the lesion lays on the ventral side of the neck or around the lower jaw / throat area, pieces of skin including the blubber can be missing in this part.	☐	☐
4	Undermining / detachment of blubber: Considerable parts of the blubber are detached from the underlying muscular tissue in large parts of the body area. Only in the areas around the caudal end of the body, the pectoral flippers, as well as the rostral part of the head, the skin and underlying tissue is often still attached.	☐	☐
5	Rake marks in blubber: Rake marks potentially as the result of the incisions by the teeth and/or claws are present in parts of the blubber tissue.	☐	☐
6	Puncture lesions: Puncture lesions are present in the skin and/or blubber tissue.	☐	☐
7	Avulsion of one or both scapulae: An avulsion of one or both scapulae due to the detachment of the skin and blubber (including the pectoral flippers) can be present.	☐	☐
8	Helical smooth edged lesion: A smooth edged laceration which follows a helical course, starting in the area of the throat / head and circling backwards once or twice around the body is a typical pattern observed.	☐	☐
9	Skeletal trauma: Fractures of bones with different severity can be present. Fractures of parts of the skull have been observed. Also puncture like fractures in the lower mandible and / or scapula have been observed commonly.	☐	☐
10	Vast amounts of soft tissue are removed: Lesions which can be situated anywhere on the body with vast amounts of skin, blubber and muscle tissue missing are especially in addition to a ragged wound margin a strong indicator for an interaction with a red fox.	☐	☐
11	Ragged wound margin (fully or in parts): Considerable parts of the wound margin have a ragged, uneven and not cut-like structure. This is a strong indication for an interaction with a red fox.	☐	☐
12	Defect of hair shafts: In contrast to torn tissue where on the cranial side of the linear wound margin largely intact hair extends over the edge of the tissue, in cut tissue the extending hair on the cranial side often shows structural damage of the hair shafts.	☐	☐

Prep.-No:______________________

Detailed morphometry:

Weight ______________________ kg

Total length (TL) ______________ cm *on back, stretched neck*

Standard length (SL) ____________ cm *on back, stretched neck*

Reduced length (RL) ____________ cm *on back, stretched neck*

Axial girth (AG) ________________ cm

Blubber thickness (breast) _________ mm *with skin*

Blubber thickness (neck) _________ mm *with skin*

Photos yes ☐ no ☐

Nutritional Status ○ good ○ moderate ○ poor

Histological samples ☐

____

____

____

Bone samples ☐

Skeleton ○

Skull ○

Other ○ ....

Notes
